# Combination antimicrobial therapy to manage resistance

**DOI:** 10.1093/emph/eoad005

**Published:** 2023-03-30

**Authors:** Robert J Woods, Andrew F Read

**Affiliations:** Department of Medicine, University of Michigan, Ann Arbor, MI, USA; Veterans Affairs Ann Arbor Healthcare System, Ann Arbor, MI, USA; Departments of Biology and Entomology, and Huck Institutes of the Life Sciences, Pennsylvania State University, University Park, PA, USA

**Keywords:** antibiotic resistance management, synergy, antagonism, off-target

## COMBINATION THERAPY: OVERVIEW

The concurrent use of multiple antimicrobials to treat patients is common in clinical practice. Combination therapy is used for many reasons, the majority of which have little to do with antimicrobial resistance [1]. However, combination therapy impacts both the likelihood and the nature of resistance evolution, thus it has been a major focus of ‘resistance management strategies’ aimed at alleviating the global antimicrobial resistance crisis.

Reasons to use combination therapy include the following: (i) increased breadth of coverage when the organism is unknown, such as when a patient initially presents with sepsis, (ii) treatment of polymicrobial infections, (iii) more efficient killing of the pathogen, such as the synergistic use of penicillin and streptomycin against Enterococci, (iv) interrupting multiple pathogenic mechanisms, such as in adding a protein synthesis inhibitor to reduce toxin production in group A Streptococcus necrotizing soft tissue infections, (v) addressing multiple anatomical compartments, (vi) slowing the elimination of an antimicrobial, as when ritonavir is added to atazanavir, (vii) lowering the dose of a single drug to reduce toxicity, as with aminoglycosides and finally (viii) preventing resistance evolution, such as in treatment of tuberculosis or HIV.

Nonetheless, combination therapy may be harmful for patients compared to monotherapy. This may arise when antimicrobials interfere with each other’s action, so called antagonism [2], or interfere with each other’s metabolism. Additionally, combination therapy can be more expensive, more complex to administer, lead to more adverse drug reactions and exacerbate loss of resistance to colonization by multidrug-resistant organisms.

## EVOLUTIONARY PERSPECTIVES

Combination therapy has been successfully used to prevent resistance evolving during the treatment of tuberculosis [3] and HIV. Resistance is likely suppressed by two processes. First, if combination therapy more effectively suppresses the population than monotherapy, the likelihood of a resistance mutation occurring, which is proportional to the population size, is reduced. Second, combination therapy further reduces the likelihood of resistance if it increases the number of simultaneous mutations required to acquire resistance or narrows the number of mutational pathways to resistance. Such strategies prevent resistance from arising in the first place.

Separately, theory and laboratory experiment show that the use of two drugs at specific doses can be used to select against resistance strains, thereby maintaining the more sensitive phenotype [4]. This has yet to be deployed in practice but is conceptually appealing because it may be used even after the resistant phenotype has arisen.

In contrast to TB and HIV, combination therapy is rarely used to prevent resistance in typical bacterial infections. This is because, for the most part, resistance evolution during treatment is not a major threat, so that monotherapy is very often successful. Additionally, the primary resistance threat when treating typical infections is not the organisms causing the infection, but commensal opportunistic pathogens harbored elsewhere in the body that are also being exposed to the antibiotic [5]. This ‘off target’ selection is occurring against the diversity of the microbiome, which is generally unknown and thus is a barrier to rational design of combinations.

## FUTURE IMPLICATIONS

The vast majority of combination therapy is given for reasons unrelated to resistance management. There is a pressing need to understand the impact that existing patterns of combination therapy have on the antibiotic resistance crisis.

The potential to develop novel combinations to manage resistance evolution has strong theoretical and experimental support. But, realizing this potential will require an understanding of the evolutionary, ecological and epidemiological complexities of combination therapy on commensal opportunistic pathogens and the broader microbiomes in numerous anatomical sites.

## Supplementary Material

eoad005_suppl_Supplementary_Data_S1

eoad005_suppl_Supplementary_Data_S2

eoad005_suppl_Supplementary_Data_S3
